# Blood pressure and hypertension in older adults with a history of regular cannabis use: findings from the Multi-Ethnic Study of Atherosclerosis

**DOI:** 10.3389/fcvm.2024.1432923

**Published:** 2024-10-30

**Authors:** Jamie Corroon, Ryan Bradley, Matthew A. Allison, Igor Grant

**Affiliations:** ^1^ Department of Family Medicine, University of California San Diego, La Jolla, CA, United States; ^2^ Herbert Wertheim School of Public Health and Human Longevity Science, University of California San Diego, La Jolla, CA, United States; ^3^ Department of Psychiatry, Center for Medicinal Cannabis Research, University of California San Diego, La Jolla, CA, United States

**Keywords:** cannabis, marijuana, blood pressure, hypertension, cardiovascular disease

## Abstract

**Background:**

Observational evidence investigating associations between cannabis use and blood pressure and hypertension is inconsistent.

**Methods:**

Cross-sectional data from 3,255 participants at Exam 6 (2016–2018) of the Multi-Ethnic Study of Atherosclerosis (MESA) were analyzed, including self-reported cannabis smoking patterns, standardized measures of systolic blood pressure (SBP), diastolic blood pressure (DBP), pulse pressure (PP; BP collectively), and hypertension. ANCOVA and multivariable relative risk regression models were used to calculate adjusted means for BP and adjusted prevalence ratios (PRs) for prevalent hypertension.

**Results:**

In fully adjusted ANCOVA models, a history of regular cannabis smoking, when compared to no history, was not significantly associated with increased SBP [mean difference: 0.1 mmHg (95% CI: −1.6–1.9)], DBP [mean difference: 0.5 mmHg (95% CI: −0.3–1.4)], PP [mean difference: −0.5 mmHg (95% CI: −1.8–0.9)], or prevalent hypertension [PR: 1.01 (95% CI: 0.93−1.10)]. Furthermore, no associations were observed for either the duration or recency (in the past month) of cannabis smoking or number of joint/pipe years. Models exploring potential interactions between a history of regular cannabis smoking and age, sex, race/ethnicity, and cigarette smoking status were not significant for either BP or hypertension.

**Conclusions:**

In a cohort of racially and ethnically diverse older adults with a high prevalence of hypertension, no evidence of increased risk due to regular cannabis smoking was found for either blood pressure or hypertension.

## Introduction

Cardiovascular effects of *Cannabis sativa* L. (cannabis, marijuana) are primarily mediated through interactions between phytocannabinoids and cannabinoid receptors (CB1R and CB2R) in the cardiovascular and nervous systems, involving both direct cardiac effects and indirect autonomic effects. These effects include increases in heart rate, cardiac contractility, and changes in vascular resistance, potentially resulting in hemodynamic changes and altered blood pressure (BP) (1). However, studies have reported significant variability in these effects (2).

Some cross-sectional studies have reported associations between recent (past month) cannabis use, frequency of recent use (days used in the past month) and elevated systolic BP (SBP) (3, 4) but not diastolic BP (DBP) or prevalent hypertension (5). Importantly these studies did not consider the duration or timing of regular use, which may result in tolerance and is more likely to underlie a chronic disease process. Questionnaires from these studies often fail to specify the method of cannabis use (inhalation, ingestion, etc.) (6), which may mediate the risk (7). Our previous investigation using data from the National Health and Nutrition Examination Survey (NHANES, 2009 and 2018) reported no associations between a history of monthly cannabis use (smoked) for more than one year, including a duration of monthly use of more than 10 years, and either increased SBP, DBP or prevalent hypertension (8). An earlier NHANES study using data from 2017 to 2018 reported similar findings for hypertension (5). Conversely, a large cross-sectional study (*n* = 91,161) utilizing data from the UK Biobank, a population-based study of around 500,000 volunteers in the UK, noted lower SBP, DBP, and pulse pressure (PP) among “heavy” lifetime cannabis users (more than 100 times in a lifetime) (9). Longitudinal research remains sparse but the available research has found no association with elevated SBP, DBP or incident hypertension (10, 11). Notably, previous studies have focused mostly on middle-aged participants. Consequently, it remains unclear whether these risks differ in older adults, who are likely to have a higher prevalence of comorbidities. This issue is particularly timely, as an increasing number of older adults' are reporting cannabis use (12).

The present study aimed to characterize cannabis smoking patterns in the Multi-Ethnic Study of Atherosclerosis (MESA) and investigate the associations between a history of regular cannabis smoking and systolic, diastolic, and pulse pressures, as well as prevalent hypertension. Based on our previous work, we hypothesized that a history of regular cannabis smoking would not be associated with elevated blood pressure or prevalent hypertension.

## Methods

### Study population

The Multi-Ethnic Study of Atherosclerosis (MESA) is a prospective cohort study of men and women aged 45–84 years who were free of clinical cardiovascular disease (CVD) at the time of study enrollment (*n* = 6,814). Participants were recruited from 2000 to 2002 in six US field centers from four race/ethnic groups of Caucasian (38%), African American (28%), Hispanic American (22%), and Chinese Americans (12%). All participants provided written informed consent. The original MESA study was approved by the institutional review board of each field center. Detailed methodology for MESA has been published previously (13).

### Analytic sample

The analytic sample (*n* = 3,255) was limited to all MESA participants with complete data for two questions used to define a history of regular cannabis use and three readings for blood pressure (SBP and DBP) at Exam 6 (2016–2018).

### Independent variables

#### Cannabis use

**Figure 1:**
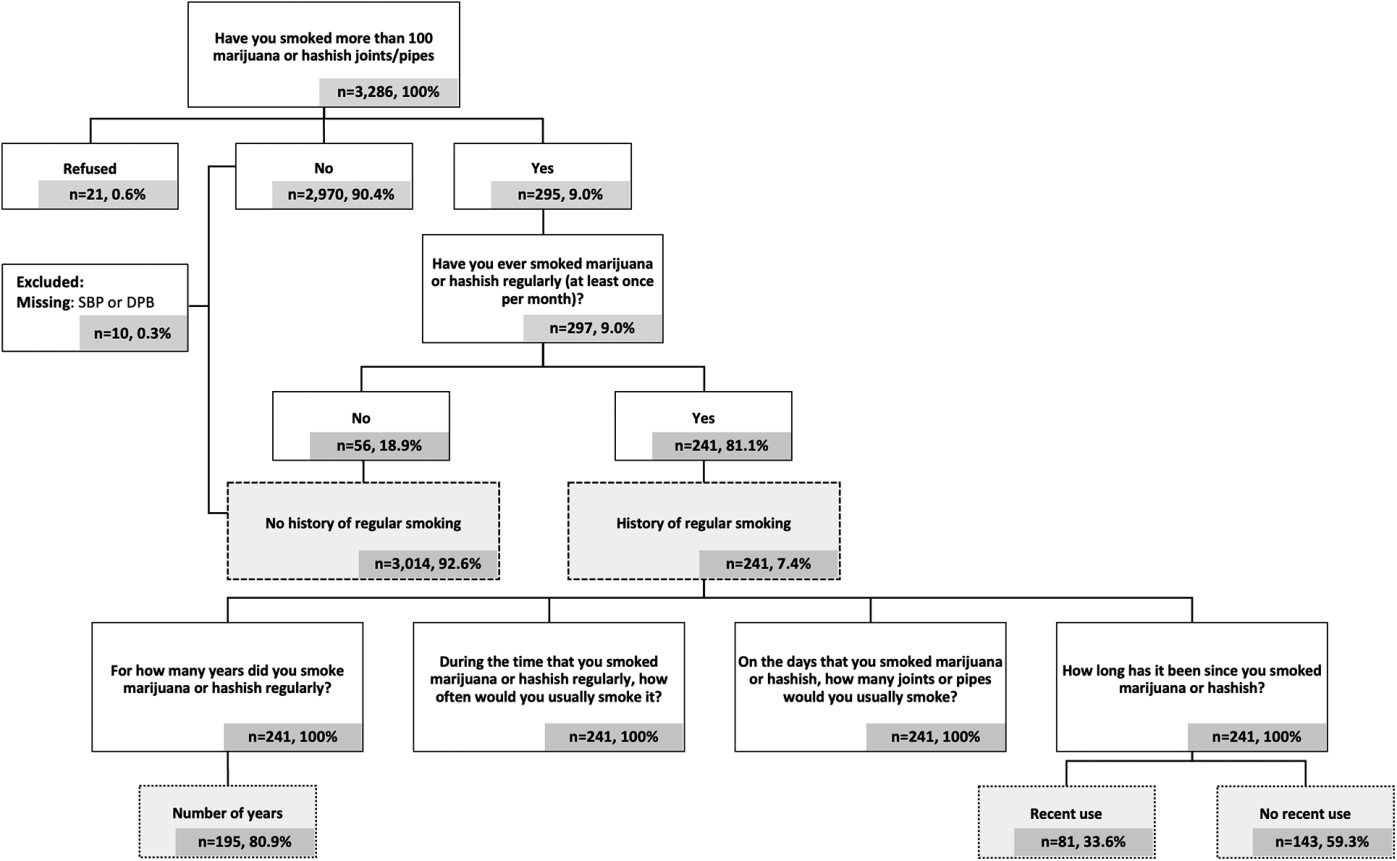
Flow diagram of study sample selection.

At Exam 6, participants responded to a series of questions about cannabis use (full questionnaire available at: https://www.mesa-nhlbi.org/PublicDocs/MESAExam6Forms/V6_PersonalHistory.pdf) (Figure 1). Participants classified as having a history of regular cannabis smoking were those who responded “Yes” to the questions: (1) “Have you smoked more than 100 marijuana or hashish joints/pipes in your life?” and (2) “Have you ever smoked marijuana or hashish regularly (at least once per month)?” Those classified as having no history of regular smoking were those who reported “Yes” or “No” to the first question and “No” to the second.

Participants with a history of regular smoking were subsequently asked, “For how many years did you smoke marijuana or hashish regularly?” and “How long has it been since you last smoked marijuana or hashish?”. Years of regular smoking were analyzed as both continuous and categorical variables. Recency of use was defined as any reported use within the preceding 31 days.

A composite measure was created (i.e., joint/pipe years) to assess a multifaceted exposure including duration, frequency, and quantity of cannabis smoking. Joint/pipe years was calculated using the low end, midpoint, and high end for each categorical response to the question “During the time that you smoked marijuana or hashish regularly, how often would you usually smoke it?” (Supplementary Methods). For the midpoint, joint/pipe years was calculated as follows: (midpoint value of each categorical response to “During the time that you smoked marijuana or hashish regularly, how often would you usually smoke it?” × “On the days that you smoked marijuana or hashish, how many joints or pipes would you usually smoke?” × 12 months/year × years of regular smoking)/days per year [For example: (6 times per month × 3 joints/pipes per day × 12 months/year × 10 years of regular smoking)/365.25 = 5.9 joint/pipe years]. Joint/pipe years was analyzed both as a continuous and dichotomous variable cut at the median of the monthly frequency.

#### Covariates

Sex, race/ethnicity, household income and educational attainment were assessed using standard questionnaires at the baseline visit (13). Age, physical activity, sedentary behavior, cigarette smoking, current alcohol use, diabetes status, and medication use were assessed using standard questionnaires at Exam 6. Dyslipidemia was defined as a total cholesterol to high density lipoprotein (HDL) ratio >5.0 or use of lipid-lowering medication. Prediabetes was defined as fasting blood glucose ≥100 mg/dl or A1c ≥5.7%. Diabetes was defined as fasting blood glucose ≥126 mg/dl, HbA1c >6.4%, or the use of antidiabetic medication. Height, weight, and biomarkers [total cholesterol (mg/dl), HDL cholesterol (mg/dl), fasting blood glucose (mg/dl), and HbA1c (%)] were objectively measured at Exam 6 in the central laboratory (University of Vermont, Burlington, VT, USA) using standardized protocols and calibrated measurements.

### Dependent variables

#### Blood pressure and hypertension

During Exam 6 (2016–2018), participants underwent standardized measurements of height, weight, blood pressure, and additional assessments. With the participant seated, resting blood pressure was measured in the morning three times with a Dinamap automated device (Model PRO 100, GE Healthcare) (average measurement time 08:54 ± 48 min). The first blood pressure reading was recorded after five minutes of sitting. Subsequent measurements were taken at five-minute intervals (13). Medical history, personal history, medication use, and physical activity were obtained using standardized questionnaires.

Three complete SBP and DBP readings were averaged to obtain mean SBP and DBP, respectively. Pulse pressure was calculated as the difference between mean SBP and DBP. Hypertension was defined as SBP ≥130 mmHg or DBP ≥80 mmHg (per the 2017 ACC/AHA Guideline for High Blood Pressure in Adults) (14) or use of antihypertensive medication. Complete BP data were available for 99.7% of respondents (*n* = 3,245).

### Statistical methods

Descriptive statistics were used to characterize the sample. Means and standard deviations were reported for continuous variables, whereas counts and percentages were used for nominal variables. Continuous variables were assessed for plausibility (Supplementary Methods).

The primary analysis tested the association between a history of regular cannabis smoking and BP and prevalent hypertension. Secondary analyses included the duration of monthly smoking and recency of smoking by those with a history of regular use.

ANCOVA models were used to estimate adjusted means and 95% confidence intervals (CIs) for SBP, DBP, and PP across cannabis smoking categories and multivariable relative risk regression models with robust standard errors were used to estimate adjusted prevalence ratios (PRs) and 95% CIs for hypertension, controlling for potential confounders. Multivariable linear regression was used in secondary analyses to evaluate the years of regular smoking and the number of joints or pipes smoked per day as continuous variables.

Variables considered as potential confounders in the multivariable analyses were sociodemographic variables including age, sex, race/ethnicity, education level, and household income (model 1); behavioral risk factors such as cigarette smoking, alcohol use, and physical activity (model 2); and BMI and CVD risk factors, including total cholesterol to high-density lipoprotein (HDL) cholesterol ratio, FBG, antilipidemic, antidiabetic, and antihypertensive medications (for SBP, DBP, and PP models only) (model 3). Assessment of collinearity between independent variables was performed in the final multivariate adjusted model using the Variance Inflation Factor and Tolerance. Statistical significance was set at *p*-value <0.05.

### Interaction

Multiplicative first-order interactions were constructed in the multivariable adjusted regression models for variables designated *a priori*: age (59–69, 70–80, >80), race/ethnicity (Non-Hispanic White people, Non-Hispanic Black people, Hispanic, Chinese), sex (male, female), and cigarette smoking status (current, former, never). Interaction terms were considered significant in the final multivariable-adjusted model if *p* < 0.10.

### Sensitivity analyses

Sensitivity analyses were performed using the aforementioned statistical approach among two subgroups: a subgroup of participants who denied a history of myocardial infarction or stroke up to and including Exam 6, and a subgroup of those not taking anti-hypertensive medications.

Data management and statistical analyses were performed using SAS On-Demand for Academics (Copyright © 2014 SAS Institute, Cary, NC, USA).

## Results

The study sample included 3,255 participants, 7.4% (*n* = 241) of whom reported a history of regular cannabis smoking of at least once per month (Table 1). The average age was 74 years (range: 59–99 years), with an almost equal distribution across sexes. Non-Hispanic White people participants comprised the largest portion of the sample (39.9%). Participants with a history of regular cannabis smoking were younger, more likely to be male, non-Hispanic Black people, consume more alcoholic drinks per week, be current cigarette smokers, and have more cigarette pack-years. In contrast, they also reported higher levels of physical activity per week and had similar BMIs.

**Table 1:** Characteristics of participants by cannabis smoking status (*n* = 3,255).

Characteristic	Overall sample	No hx of regular cannabis smoking	Hx of regular cannabis smoking
	(*n* = 3,255, 100.0%)	(*n* = 3,014, 92.6%)	(*n* = 241, 7.4%)
Socio-demographics			
Age (years), mean (sd)	74.2 (8.7)	74.8 (8.7)	67.3 (5.4)
Male, *n* (col. %)	1,520 (46.7)	1,351 (44.8)	169 (70.1)
Race/Ethnicity, *n* (col. %)			
Non-Hispanic White people	1,299 (39.9)	1,192 (39.6)	107 (44.4)
Non-Hispanic Black people	833 (25.6)	728 (24.2)	105 (43.6)
Hispanic	704 (21.6)	676 (22.4)	28 (11.6)
Chinese	419 (12.9)	418 (13.9)	1 (0.4)
Education, *n* (col. %)			
<Bachelor's degree	1,889 (58.2)	1,762 (58.6)	127 (52.7)
Bachelor's degree	617 (19.0)	563 (18.7)	54 (22.4)
Graduate or Professional School	742 (22.8)	682 (22.7)	60 (24.9)
Gross Family Income, *n* (col. %)			
≤$49,999	1,600 (51.4)	1,510 (52.5)	90 (38.1)
$50,000–$99,999	862 (27.7)	776 (27.0)	86 (36.4)
≥$100,000	651 (20.9)	591 (20.5)	60 (25.4)
Lifestyle, behavioral			
Alcohol intake (drinks/week), mean (sd)	5.8 (7.8)	5.5 (7.3)	7.9 (10.9)
Smoking status, *n* (col. %)			
Never	1,507 (46.3)	1,462 (48.5)	45 (18.7)
Former	1,567 (48.1)	1,406 (46.7)	161 (66.8)
Current	181 (5.6)	146 (4.8)	35 (14.5)
Smoking (pack years), mean (sd)	10.0 (19.4)	9.3 (19.0)	18.0 (22.4)
Physical activity (met-min/week), mean (sd)	4,906 (5,222)	4,715 (5,075)	7,194 (6,314)
BMI, mean (sd)	28.4 (5.7)	28.4 (5.8)	28.6 (5.4)
Cardiometabolic medical history			
Systolic BP (mmHg), mean (sd)	128 (21)	129 (21)	125 (20)
Diastolic BP (mmHg), mean (sd)	69 (10)	69 (10)	72 (11)
Pulse pressure (mmHg), mean (sd)	59 (17)	60 (17)	52 (15)
Hypertension, *n* (col. %)	2,450 (75.3)	2,279 (75.6)	171 (71.0)
Dyslipidemia, *n* (col. %)	1,705 (52.4)	1,595 (52.9)	110 (45.6)
Diabetes or prediabetes, *n* (col. %)	1,807 (55.5)	1,682 (55.8)	125 (51.9)
Medications, *n* (col. %)			
Antihypertensive	1,996 (61.3)	1,869 (62.0)	127 (52.7)
Antilipidemic	1,532 (47.1)	1,432 (47.5)	100 (41.5)
Antidiabetic	618 (19.0)	587 (19.5)	31 (12.9)
Total medications, mean (sd)	7.1 (4.7)	7.1 (4.6)	7.1 (5.4)

Hx, history; sd, standard deviation; BMI, body mass index; met-min/week, metabolic equivalent of task minutes per week; mmHg, millimeters mercury.

Almost 13% of non-Hispanic Black people participants reported a history of regular cannabis smoking (Table 2), which was higher than 8.2% of non-Hispanic White people participants and 4.0% of Hispanic participants. The duration of regular smoking was also the highest among non-Hispanic Black people participants, at almost 20 years on average, compared to almost 14 years for non-Hispanic White people participants and almost 15 years for Hispanic participants. Among these groups, Hispanic participants had the highest percentage of participants who smoked cannabis daily (42.9%). Use in the past month was reported by 33.6% of participants with a history of regular cannabis smoking. Data for Chinese participants were limited, as only one participant reported a history of regular smoking.

**Table 2:** Cannabis smoking characteristics of participants with a history of regular use by race/ethnicity (*n* = 241).

Characteristic	Overall Sample	Non-Hispanic White people	Non-Hispanic Black people	Hispanic	Chinese
	(*n* = 241, 100.0%)	(*n* = 107, 44.4%)	(*n* = 105, 43.6%)	(*n* = 28 11.6%)	(*n* = 41, 0.4%)
History of regular smoking (yes), *n* (col. %)					
Among all participants	241 (7.4)	107 (8.2)	105 (12.6)	28 (4.0)	1 (0.2)
Among those with ≥100 Joints/pipes lifetime	241 (81.1)	107 (81.7)	105 (81.4)	28 (80.0)	1 (50.0)
Duration of regular smoking, mean (sd)					
Years (*n* = 195)	16.1 (14.8)	13.9 (13.7)	19.4 (16.2)	14.7 (12.2)	5.0 (0.0)
Frequency, *n* (col. %)					
<Once a week	35 (14.5)	16 (15.0)	13 (12.4)	6 (21.4)	0 (0.0)
≥Once a week	130 (53.9)	64 (59.8)	56 (53.3)	9 (32.1)	1 (100.0)
Daily	63 (26.1)	23 (21.5)	28 (26.7)	12 (42.9)	0 (0.0)
Missing/Refused/Don't know	13 (5.4)	4 (3.7)	8 (7.6)	1 (3.6)	0 (0.0)
Quantity of daily use, *n* (col. %)					
≤1 joints/pipes	102 (42.3)	56 (52.3)	37 (35.2)	9 (32.1)	0 (0.0)
>1–3 joints/pipes	60 (24.9)	18 (16.8)	34 (32.4)	8 (28.6)	0 (0.0)
>3 joints/pipes	21 (8.7)	7 (6.5)	10 (9.5)	4 (14.3)	0 (0.0)
Missing/Refused/Don't know	58 (24.1)	26 (24.3)	24 (22.9)	7 (25.0)	1 (100.0)
Duration, frequency, and quantity, median (iqr)					
Joint/pipe years (midpoint, *n* = 228)	8.4 (22.6)	5.0 (15.3)	10.8 (46.4)	6.9 (21.7)	0 (0.0)
Recency, *n* (col. %)					
Past month use (*n* = 224)					
Among all participants	81 (2.5)	40 (3.1)	37 (4.4)	4 (0.6)	0 (0.0)
Among those with history of regular smoking	81 (33.6)	40 (37.4)	37 (35.2)	4 (14.3)	0 (0.0)

sd, standard deviation.

### Blood pressure

In unadjusted ANOVA models, a history of regular cannabis smoking, when compared to no history, was associated with lower SBP [mean difference: −4 mmHg (95% CI: −1 to −7), *p* < 0.01] and PP [−8 mmHg (95% CI: −6 to −10), *p* < 0.01], but higher DBP [4 mmHg (95% CI: 2–5), *p* < 0.01] (Table 3A). However, in fully adjusted ANCOVA models, the differences were attenuated and no longer significant (Table 3A).

**Table 3A:** Adjusted mean blood pressure (mmHg) and 95% confidence intervals by cannabis smoking status.

Characteristic	Unadjusted			Model 3		
	SBP	DBP	PP	SBP	DBP	PP
History of regular smoking	(*n* = 3,255)			(*n* = 2,824)		
No	129 (128–129)	69 (68–69)	60 (59–60)	127 (125–128)	68 (68–69)	58 (57–59)
Yes	125 (122–127)	72 (71–74)	52 (50–54)	127 (124–130)	69 (68–70)	57 (55–60)
*p*	**<.01**	**<.01**	**<.01**	0.93	0.48	0.67
Duration of regular smoking, years	(*n* = 3,210)			(*n* = 2,785)		
0	129 (128–129)	69 (68–69)	60 (59–60)	127 (125–128)	68 (68–69)	58 (57–59)
1–5	124 (119–129)	72 (69–74)	52 (48–56)	127 (122–132)	69 (66–71)	58 (54–62)
>5–10	123 (117–129)	72 (69–75)	50 (45–56)	125 (118–131)	67 (64–70)	57 (52–62)
>10	124 (120–129)	72 (70–74)	52 (48–56)	127 (122–132)	70 (67–72)	57 (54–61)
*p*	**0.03**	**<.01**	**<.01**	0.93	0.66	0.96
Recency of smoking	(*n* = 3,239)			(*n* = 2,809)		
No history of regular smoking	129 (128–129)	69 (68–69)	60 (59–60)	127 (125–128)	68 (68–69)	58 (57–59)
No past month smoking	124 (120–127)	72 (70–73)	52 (49–55)	126 (122–129)	68 (67–70)	57 (54–60)
Past month smoking	125 (120–130)	73 (71–75)	52 (48–55)	128 (123–132)	69 (67–72)	58 (54–62)
*p*	**0.01**	**<.01**	**<.01**	0.73	0.72	0.83

All comparisons include participants who reported no history of regular smoking.

Model 3: age, gender, race/ethnicity, HH income, alcohol and cigarette use, physical activity, BMI, total cholesterol to HDL cholesterol ratio, FBG, antihypertensive, antilipidemic, and antidiabetic medications.

*p*-values for multiple comparisons have been adjusted using Tukey's method.

Bold = *p* < 0.05.

**Figure 2:**
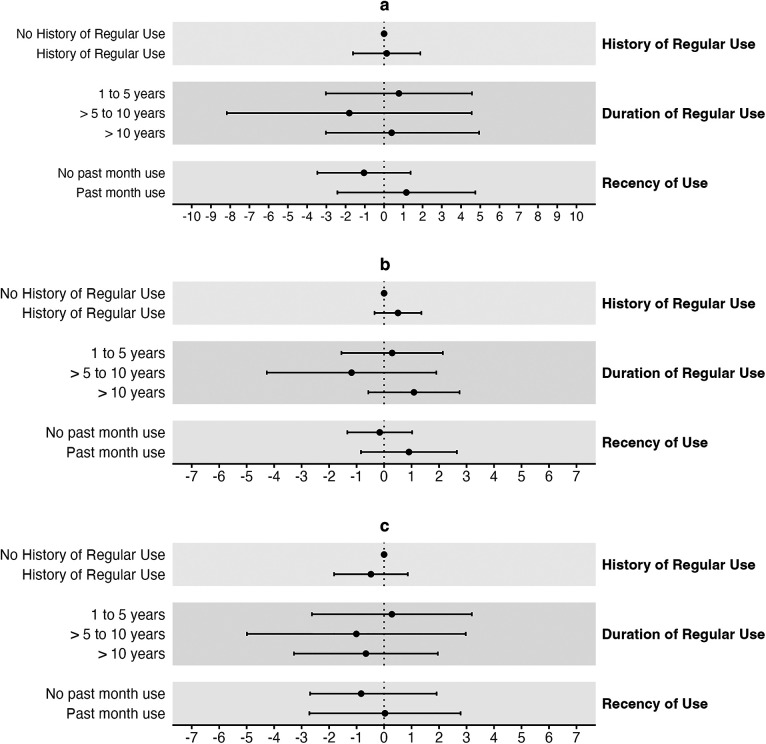
Adjusted mean differences for blood pressure by cannabis smoking status. **(a)** Adjusted mean differences and 95% CIs for SBP (mmHg) by cannabis smoking status. **(b)** DBP (mmHg). **(c)** PP (mmHg). Each grey band represents a separate model with a unique exposure variable, but identical covariates and outcome variable. All comparisons are made to individuals with no history of regular cannabis smoking. Cls: confidence intervals.

The duration of regular cannabis smoking was also associated with lower SBP and PP but higher DBP in unadjusted models (Table 3A). However, in fully adjusted ANCOVA models, the differences were attenuated and no longer significant (Table 3A, Figures 2a–c).

When analyzed continuously in fully adjusted multivariable linear regression models, each additional year of regular cannabis smoking was not associated with SBP [*β* = 0.0 mmHg (95% CI: −0.1–0.2)], DBP [*β* = 0.0 mmHg (95% CI: −0.1–0.1)] or PP [*β* = 0.0 mmHg (95% CI: −0.1–0.1)] (Supplementary Table S4). Similarly, the number of joint or pipe years was also not associated with any of the blood pressure outcomes at the low end, midpoint, or high end of monthly frequency (midpoint: SBP: *β* = 0.0 mmHg (95% CI: 0.0–0.1); DBP: *β* = 0.0 mmHg (95% CI: 0.0–0.0); PP: *β* = 0.0 mmHg (95% CI: 0.0–0.1) (Supplementary Table S4).

In unadjusted ANOVA models, and when compared to no history, cannabis smoking in the past month (recency) by participants with a history of regular smoking was associated with lower SBP (*p* = 0.01) and PP (*p* < 0.01), but higher DBP (*p* < 0.01) (Table 3A). However, in fully adjusted ANCOVA models, the differences were attenuated and no longer significant (Table 3A). Further, no significant differences in SBP (*p* = 0.77), DBP (*p* = 0.49), or PP (*p* = 0.78) were observed in fully adjusted ANCOVA models when participants who reported a history of regular smoking *and* smoking in the past month were compared to those who reported no smoking in the past month.

### Hypertension

In both unadjusted and adjusted multivariable relative risk regression models, neither a history of regular cannabis smoking nor the duration nor recency of smoking was associated with hypertension prevalence (Table 3B).

**Table 3B:** Adjusted prevalence ratios and 95% confidence intervals for hypertension by cannabis smoking status.

Characteristic	Unadjusted	Model 3
	PR (95% CI)	
History of regular smoking	(*n* = 3,255)	(*n* = 2,824)
No	**1.00 Ref.**	**1.00 Ref.**
Yes	0.94 (0.86–1.02)	1.04 (0.97–1.11)
Duration of regular smoking, years	(*n* = 3,210)	(*n* = 2785)
0	**1.00 Ref.**	**1.00 Ref.**
1 to 5	0.91 (0.77–1.07)	1.01 (0.87–1.18)
> 5 to 10	0.93 (0.77–1.13)	0.98 (0.86–1.12)
> 10	0.91 (0.79–1.06)	0.97 (0.78–1.21)
Recency	(*n* = 3,239)	(*n* = 2809)
No history of regular smoking	**1.00 Ref.**	**1.00 Ref.**
No past month smoking	0.96 (0.87–1.07)	0.99 (0.89–1.10)
Past month smoking	0.88 (0.75–1.03)	1.01 (0.86–1.17)

Model 3: age, gender, race/ethnicity, HH income, alcohol and cigarette use, physical activity, BMI, total cholesterol to HDL cholesterol ratio, FBG, antihypertensive, antilipidemic, and antidiabetic medications.

CI, confidence interval; HH, household; BMI, body mass index; HDL, high density lipoprotein; FBG, fasting blood glucose.

Bold = *p* < 0.05.

Similarly, when analyzed in the fully adjusted relative risk regression model as a dichotomous variable divided at the median, joint/pipe years was not associated with the prevalence of hypertension at the low end, midpoint, or high end of monthly frequency (Supplementary Table S5).

### Interaction effects

Models exploring potential interactions between a history of regular cannabis smoking and age, sex, race/ethnicity, and cigarette smoking status were not significant for either BP or hypertension (Supplementary Tables S3A, S3B).

### Sensitivity analyses

To evaluate the stability and reliability of the findings, sensitivity analyses were conducted on two subgroups of participants: (1) those who had not undergone any interventions for myocardial infarction or stroke (*n* = 3,015) up to and including Exam 6 (*n* = 240 for both); and (2) those who did not report taking anti-hypertensive medication (*n* = 1,259).

### Subgroup analyses in those not reporting myocardial infarction or stroke

In fully adjusted ANCOVA models, among participants without prior interventions for myocardial infarction or stroke, no associations were observed between a history of regular cannabis smoking, duration of smoking, or recency of smoking and BP when compared to those with no history (Supplementary Table S6). Similarly, no associations with BP were observed when participants who reported recent smoking were compared to those who denied recent it.

No associations were observed with hypertension (Supplementary Table S7).

### Subgroup analyses in those not taking anti-hypertensive medications

Results for participants who denied the use of pharmacological interventions for blood pressure control were similar to those without prior interventions for myocardial infarction or stroke (Supplementary Tables S8, S9).

## Discussion

In a cohort of racially and ethnically diverse older adults with a high prevalence of hypertension, no associations were found between a history of regular cannabis smoking, duration, or recency of smoking, and either SBP, DBP, or PP, or the prevalence of hypertension. None of these associations were modified by factors such as age, sex, race/ethnicity, or cigarette smoking. In addition, the absence of associations found in the overall sample was largely mirrored in two subgroups: those without a history of either myocardial infarction or stroke and those not taking anti-hypertensive medications. Therefore, these results remain robust despite the possibility of increased medical intervention.

The findings of this study align with our previous results from NHANES (8) and corroborate evidence from other cross-sectional studies investigating regular cannabis use (5), including a large cross-sectional study (*n* = 91,161) utilizing data from the UK Biobank, which reported an association between more than 100 uses of cannabis in a lifetime and lower SBP, DBP and PP, when compared to never use (method of use unspecified) (9). Unlike that study however, where lower blood pressure was reported among women as compared to men, we did not observe a significant interaction by sex (*p* for interaction = 0.22).

Longitudinal studies have also reported a lack of association between lifetime cannabis use and increased blood pressure (10) and the incidence of hypertension (11). Taken together, these studies suggest that regular cannabis use is not associated with elevated blood pressure or hypertension.

Notably, despite differences in chemical composition and patterns of use, cannabis smoke contains many of the same toxic byproducts of combustion as tobacco smoke (15, 16). Like tobacco smoke, cannabis smoke has been associated with endothelial dysfunction, arterial stiffness, and acute increases in blood pressure (16). Despite this, epidemiological studies have reported mixed associations with elevated blood pressure and hypertension for both substances (17–19). For cannabis, the modulation of the endocannabinoid system by phytocannabinoids (20), may mitigate adverse impacts on the cardiovascular system which may otherwise lead to increases in blood pressure.

In MESA, more than 9% (*n* = 295) of participants reported smoking 100 or more joints or pipes in their lifetime, and 7.4% (*n* = 241) reported a history of regular smoking at a frequency of at least once per month. This compares to 4.4% who reported more than 100 lifetime uses in the UK Biobank (9) and 25.5% of NHANES respondents who reported a history of regular smoking for more than one year in our previous publication on this topic. Differences in exposure across these samples may be explained by sex and race/ethnicity. The NHANES sample comprised a greater proportion of the most likely subgroup to report a history of regular use (non-Hispanic White people males; *n* = 1,828, 18.7%). In the present study, non-Hispanic Black people males, who were the most likely to report a history of regular smoking, comprised a smaller proportion of the sample (*n* = 354, 10.9%). The UK Biobank sample lacked information on race/ethnicity and age range. Additionally, the age ranges of the samples varied, with minimal overlap in the distribution (MESA: 59–99 years; NHANES: 35–59; and UK Biobank: –51), although this difference is more likely to influence the duration of regular use, as opposed to a history of regular use (yes/no). Further comparison of the MESA and NHANES samples showed that the mean duration of use was similar, with 16.1 years (SD = 14.8) for MESA vs. 16.5 years (SE = 0.4) for NHANES.

In contrast to much of the existing evidence indicating that cannabis users typically report lower income (21–23), our study found a significant positive association between a history of regular cannabis smoking and higher family income (χ^2^; *p* = 0.0001). This unexpected finding may reflect changes in the social and economic demographics of cannabis users, potentially influenced by the evolving legal landscape and the increasing acceptance of cannabis use across various socioeconomic strata. Alternatively, the observed association may be driven by underreporting of cannabis use among lower-income participants due to stigma or concerns about legal repercussions.

The strengths of our study include the use of a racially and ethnically diverse sample, a relatively large sample size, a specified method of cannabis use (smoking) and the use of a standardized objective outcome measure. The focus on history of regular smoking, including the duration and recency of smoking, is also a strength, given that repeated exposure over time, rather than recent use, is likely to be the main contributor to chronic cardiovascular disease. Limitations include the cross-sectional design, self-reported exposure, which could be exacerbated by the impact of long-term cannabis use on memory (24), and the relatively small number of MESA participants who reported a history of regular smoking (*n* = 241), which may limit the statistical power. In addition, other measures of cannabis exposure were not available in the dataset to include the actual amount of cannabis (grams of dried flower or extracted oil) and/or cannabinoids [milligrams of phytocannabinoids like Delta-9-tetrahydrocannabinol (THC) and cannabidiol (CBD)].

Future observational studies should aim to further explore causality using longitudinal data and to investigate potential differences attributable to the method of cannabis use, specifically comparing combusted (smoked) vs. non-combusted forms, as well as differences between inhaled and non-inhaled routes of administration (25).

Our results highlight the importance of understanding cannabis use patterns in both public health surveillance and clinical practice, especially as global trends indicate an increasing prevalence of cannabis use among older populations, a demographic already at higher risk for cardiovascular diseases.

## Conclusion

In a cohort of racially and ethnically diverse older adults with a high prevalence of hypertension, no evidence of increased risk due to regular cannabis smoking was found for either blood pressure or hypertension.

## Data Availability

The data analyzed in this study is subject to the following licenses/restrictions: MESA data is available pursuant to an approved proposal by the MESA Paper and Proposal Committee. Requests to access these datasets should be directed to https://internal.mesa-nhlbi.org.
