# Supplementary material for: Blood pressure and hypertension in older adults with a history of regular cannabis use: findings from the Multi-Ethnic Study of Atherosclerosis
Source: Front Cardiovasc Med. 2024 Oct 30;11:1432923. doi: 10.3389/fcvm.2024.1432923 (PMC11557345; doi:10.3389/fcvm.2024.1432923)
Supplement: Supplementary file 1 [file Datasheet1.pdf]

SUPPLEMENTARY MATERIAL: Blood Pressure and Hypertension in Older Adults with a History of Regular Cannabis Use: Findings from the Multi-Ethnic Study of Atherosclerosis (MESA)

Jamie Corroon, ND, MPH[1]
Ryan Bradley, ND, MPH[2]
Matthew A Allison, MD, MPH, FAHA[1]
Igor Grant MD, FRCP(C)[3]

[1] Department of Family Medicine, University of California San Diego, La Jolla, CA.
[2] Herbert Wertheim School of Public Health and Human Longevity Science, University of California San Diego, La Jolla, CA.
[3] Center for Medicinal Cannabis Research, Department of Psychiatry, University of California San Diego, La Jolla, CA.

Corresponding author: Jamie Corroon, ND, MPH (jcorroon@health.ucsd.edu)

Table of Contents

SUPPLEMENTARY METHODS 2
Composite exposure 2
Covariates 2
Interaction analysis 2
SUPPLEMENTARY RESULTS 3
Table 1 (Supplementary) - Adjusted mean blood pressure and 95% confidence intervals by cannabis smoking status 3
Table 2 (Supplementary) - Adjusted prevalence ratios and 95% confidence intervals for hypertension by cannabis smoking status 5
Table 3a (Supplementary) – Adjusted mean blood pressure for a history of regular cannabis smoking, stratified by selected characteristics 6
Table 3b (Supplementary) - Adjusted prevalence ratios and 95% confidence intervals for hypertension, stratified by selected characteristics 7
Table 4 (Supplementary) – Adjusted beta coefficients for blood pressure for selected cannabis smoking characteristics 8
Table 5 (Supplementary) – Adjusted mean blood pressure and 95% confidence intervals among participants denying MI or stroke, by cannabis smoking status (subgroup, n=3,105) 9
Table 6 (Supplementary) - Adjusted prevalence ratios and 95% confidence intervals for hypertension among participants denying MI or stroke, by cannabis smoking status (subgroup, n=3,105) 10
Table 7 (Supplementary) – Adjusted mean blood pressure and 95% confidence intervals among participants not taking anti-hypertensive medications, by cannabis smoking status (subgroup, n=1,259) 11
Table 8 (Supplementary) - Adjusted prevalence ratios and 95% confidence intervals for hypertension among participants not taking anti-hypertensive medications, by cannabis smoking status (subgroup, n=1,259) 12
References 13

SUPPLEMENTARY METHODS

Composite exposure

A composite measure was created (i.e., joint/pipe years) to assess a multifaceted exposure including duration, frequency, and quantity of cannabis smoking. Joint/pipe years was calculated using the low end, midpoint, and high end for each categorical response to “During the time that you smoked marijuana or hashish regularly, how often would you usually smoke it?”. For the midpoint, joint/pipe years was calculated as follows: (midpoint value of each categorical response to “During the time that you smoked marijuana or hashish regularly, how often would you usually smoke it?” x 12 months/year x “On the days that you smoked marijuana or hashish, how many joints or pipes would you usually smoke?” x years of regular smoking) / days per year (For example: (6 times per month x 12 months/year x 3 joints/pipes per day x 10 years of regular smoking) / 365.25 = 5.9 joint/pipe years. Midpoint values were determined as follows: “Missing”=0, “once per month” =1, “2-3 time per month” =2.5, “4-8 times per month (about 1-2 times per week)”=6, “9-24 times per month (about 3-5 times per week)”=16.5, “25-30 times per month (about one or more times per day)” =27.5.

Covariates

Sex, race/ethnicity, household income and educational attainment were assessed using standard questionnaires at the baseline visit.(1) Age, physical activity, cigarette smoking, current alcohol use, diabetes status, and medication use were assessed using standard questionnaires at Exam 6. Physical activity was reported as the total of moderate and vigorous activity, measured in MET-min/week from Monday through Sunday. Values exceeding the 99th percentile (n=31) were deemed unrealistic and were set equal to the 99th percentile (27,870 MET-min/week).

Interaction analysis

Multiplicative, first-order interactions were constructed in the multivariable adjusted regression models for variables designated a priori: age (59-69, 70-80, > 80), race/ethnicity (Non-Hispanic White, Non-Hispanic Black, Hispanic, Chinese), sex (male, female), and cigarette smoking status (current, past, never). Interaction terms were considered significant in the final multivariable adjusted model if $p < 0.10$.

SUPPLEMENTARY RESULTS

Table 1 (Supplementary) - Adjusted mean blood pressure and 95% confidence intervals by cannabis smoking status

Characteristic	Unadjusted			Model 1			Model 2			Model 3		
	**SBP**	**DBP**	**PP**	**SBP**	**DBP**	**PP**	**SBP**	**DBP**	**PP**	**SBP**	**DBP**	**PP**
**≥ 100 Joints/pipes lifetime**												
No	129 (128-129)	69 (68-69)	60 (59-61)	127 (126-128)	69 (68-69)	58 (57-58)	126 (124-128)	70 (69-71)	56 (55-57)	127 (125-128)	68 (68-69)	58 (57-59)
Yes	125 (122-127)	72 (71-73)	53 (51-54)	126 (124-129)	69 (68-71)	56 (54-58)	126 (123-130)	69 (68-71)	56 (54-59)	126 (124-129)	69 (67-70)	57 (55-60)
**p**	**<.01**	**<.01**	**<.01**	0.61	0.40	0.18	0.83	0.70	0.80	0.88	0.78	0.63
**History of regular smoking**												
No	129 (128-129)	69 (68-69)	60 (59-60)	127 (126-128)	69 (68-69)	58 (57-58)	126 (124-128)	70 (69-71)	56 (55-57)	127 (125-128)	68 (68-69)	58 (57-59)
Yes	125 (122-127)	72 (71-74)	52 (50-54)	126 (124-129)	70 (0-71)	56 (54-58)	126 (122-130)	70 (68-72)	56 (53-58)	127 (124-130)	69 (68-70)	57 (55-60)
**p**	**<.01**	**<.01**	**<.01**	0.75	0.16	0.14	0.95	0.85	0.76	0.93	0.48	0.67
**Duration of regular smoking, years**												
0	129 (128-129)	69 (68-69)	60 (59-61)	127 (126-128)	69 (68-69)	58 (57-58)	126 (124-128)	70 (69-71)	56 (55-57)	127 (125-128)	68 (68-69)	58 (57-59)
1 to 5	124 (119-129)	72 (69-74)	52 (48-56)	129 (124-134)	70 (67-72)	58 (55-62)	126 (120-132)	69 (66-72)	56 (51-60)	127 (122-132)	69 (66-71)	58 (54-62)
> 5 to 10	123 (117-129)	72 (69-75)	50 (45-56)	124 (118-130)	69 (66-72)	54 (49-59)	122 (114-130)	67 (63-71)	53 (47-60)	125 (118-131)	67 (64-70)	57 (52-62)
> 10	124 (120-129)	72 (70-74)	52 (48-56)	126 (121-130)	70 (68-72)	56 (52-59)	127 (121-133)	71 (68-73)	56 (52-61)	127 (122-132)	70 (67-72)	57 (54-61)
**p**	**0.03**	**<.01**	**<.01**	0.59	0.75	0.26	0.77	0.55	0.86	0.93	0.66	0.96
**Frequency of regular smoking**												
0 times a week	129 (128-129)	69 (68-69)	60 (59-61)	127 (126-128)	69 (68-69)	58 (57-58)	126 (124-128)	70 (69-71)	56 (55-57)	127 (125-128)	68 (68-69)	58 (57-59)
< Once a week	125 (118-132)	71 (68-74)	54 (48-60)	126 (119-133)	70 (67-73)	56 (51-61)	129 (121-138)	72 (67-76)	58 (51-64)	128 (121-135)	70 (66-73)	58 (53-63)
≥ Once a week	124 (120-127)	73 (71-75)	51 (48-53)	126 (123-130)	70 (68-72)	56 (53-59)	126 (122-131)	70 (68-72)	56 (52-59)	127 (123-130)	69 (67-71)	57 (55-60)
Daily	125 (120-130)	72 (69-74)	53 (48-57)	126 (121-131)	69 (67-72)	57 (53-61)	124 (117-130)	68 (65-71)	55 (50-60)	126 (121-132)	68 (65-71)	58 (54-62)
**p**	**0.03**	**<.01**	**<.01**	0.99	0.49	0.59	0.73	0.49	0.93	0.99	0.72	0.99
**Quantity of daily smoking**												
0 joints/pipes	129 (128-129)	69 (68-69)	60 (59-61)	127 (126-128)	69 (68-69)	58 (57-58)	126 (124-128)	70 (69-71)	56 (55-57)	127 (125-128)	68 (68-69)	58 (57-59)
≤ 1 joints/pipes	125 (121-129)	73 (71-75)	51 (48-54)	128 (124-132)	71 (69-73)	56 (53-59)	128 (123-133)	71 (69-74)	56 (53-60)	128 (124-133)	71 (69-73)	57 (54-61)
1 to 3 joints/pipes	126 (121-132)	71 (69-74)	55 (50-59)	126 (121-131)	68 (66-71)	58 (54-62)	127 (120-134)	69 (66-73)	58 (52-63)	127 (121-132)	67 (65-70)	59 (55-64)

> 3 joints/pipes	126 (117-135)	75 (71-79)	51 (44-59)	127 (118-136)	71 (67-76)	56 (49-63)	126 (114-138)	70 (65-76)	56 (47-65)	126 (118-135)	71 (66-75)	56 (50-63)
**p**	**0.31**	**<.01**	**<.01**	0.93	**0.03**	0.70	0.81	0.61	0.95	0.83	0.09	0.81
**Recency of smoking**												
No history of regular smoking	129 (128-129)	69 (68-69)	60 (59-61)	127 (126-128)	69 (68-69)	58 (57-58)	126 (124-128)	70 (69-71)	56 (55-57)	127 (125-128)	68 (68-69)	58 (57-59)
No past month smoking	124 (120-127)	72 (70-73)	52 (49-55)	126 (122-129)	69 (67-71)	56 (54-59)	125 (120-130)	69 (66-71)	56 (52-59)	126 (122-129)	68 (67-70)	57 (54-60)
Past month smoking	125 (120-130)	73 (71-75)	52 (48-55)	126 (122-131)	70 (68-72)	56 (52-59)	127 (122-132)	71 (68-73)	56 (52-60)	128 (123-132)	69 (67-72)	58 (54-62)
**p**	**0.01**	**<.01**	**<.01**	0.82	0.43	0.33	0.85	0.52	0.98	0.73	0.72	0.83

All comparisons include participants who reported no history of regular smoking.
Model 1: age, gender, race/ethnicity, HH income.
Model 2: Model 1 plus alcohol and cigarette use, physical activity
Model 3 : Model 2 plus BMI, total cholesterol to HDL cholesterol ratio, FBG, antihypertensive, antilipidemic, and antidiabetic medications.
Abbreviations: CI, confidence interval; HH, household; BMI, body mass index; HDL, high density lipoprotein; FBG, fasting blood glucose
p-values for multiple comparisons have been adjusted using Tukey's method.
The largest significant p value was reported when multiple significant differences were found.
Bold = p < 0.05

Table 2 (Supplementary) - Adjusted prevalence ratios and 95% confidence intervals for hypertension by cannabis smoking status

Characteristic	Unadjusted	Model 1	Model 2	Model 3
		PR (95% CI)		
**≥ 100 Joints/pipes lifetime**				
No	**1.00 Ref.**	**1.00 Ref.**	**1.00 Ref.**	**1.00 Ref.**
Yes	0.93 (0.86-1.01)	0.97 (0.90-1.05)	0.98 (0.90-1.06)	0.98 (0.91-1.06)
**History of regular smoking**				
No	**1.00 Ref.**	**1.00 Ref.**	**1.00 Ref.**	**1.00 Ref.**
Yes	0.94 (0.86-1.02)	1.00 (0.92-1.09)	1.00 (0.92-1.10)	1.01 (0.93-1.10)
**Duration of regular smoking, years**				
0	**1.00 Ref.**	**1.00 Ref.**	**1.00 Ref.**	**1.00 Ref.**
1 to 5	0.91 (0.77-1.07)	1.04 (0.89-1.23)	1.03 (0.87-1.21)	1.01 (0.87-1.18)
> 5 to 10	0.93 (0.77-1.13)	0.97 (0.81-1.18)	0.93 (0.74-1.16)	0.98 (0.86-1.12)
> 10	0.91 (0.79-1.06)	0.95 (0.83-1.10)	0.97 (0.85-1.11)	0.97 (0.78-1.21)
**Frequency of regular smoking**				
0 times per week	**1.00 Ref.**	**1.00 Ref.**	**1.00 Ref.**	**1.00 Ref.**
< Once a week	0.98 (0.81-1.20)	1.08 (0.89-1.31)	1.09 (0.90-1.33)	1.09 (0.91-1.30)
≥ Once a week	0.91 (0.80-1.02)	0.98 (0.87-1.11)	0.99 (0.88-1.11)	1.01 (0.90-1.14)
Daily	0.97 (0.83-1.12)	1.00 (0.86-1.16)	0.98 (0.84-1.15)	0.97 (0.84-1.14)
**Quantity of daily smoking**				
0 joints/pipes	**1.00 Ref.**	**1.00 Ref.**	**1.00 Ref.**	**1.00 Ref.**
≤ 1 joints/pipes	0.96 (0.84-1.10)	0.98 (0.86-1.12)	0.97 (0.85-1.11)	1.04 (0.91-1.18)
> 1 joints/pipes	0.89 (0.78-1.02)	0.99 (0.87-1.13)	1.01 (0.88-1.15)	0.98 (0.85-1.12)
**Recency of smoking**				
No history of regular smoking	**1.00 Ref.**	**1.00 Ref.**	**1.00 Ref.**	**1.00 Ref.**
No past month smoking	0.96 (0.87-1.07)	1.02 (0.92-1.13)	1.00 (0.90-1.11)	0.99 (0.89-1.10)
Past month smoking	0.88 (0.75-1.03)	0.94 (0.81-1.10)	0.98 (0.84-1.14)	1.01 (0.86-1.17)

Model 1: age, gender, race/ethnicity, HH income.
Model 2: Model 1 plus alcohol and cigarette use, physical activity
Model 3 : Model 2 plus BMI, total cholesterol to HDL cholesterol ratio, FBG, antilipidemic, and antidiabetic medications.
Abbreviations: CI, confidence interval; HH, household; BMI, body mass index; HDL, high density lipoprotein; FBG, fasting blood glucose
Bold = p < 0.05

Table 3a (Supplementary) – Adjusted mean blood pressure for a history of regular cannabis smoking, stratified by selected characteristics

**Characteristic**	**Age (n=2,824)**				**Sex (n=2,824)**			**Race/Ethnicity (n=2,824)**					**Tobacco Smoking Status (n=2,857)**			
	**59-69**	**70-80**	**> 80**	**p**	**Male**	**Female**	**p**	**Non-Hispanic White**	**Non-Hispanic Black**	**Hispanic**	**Chinese**	**p**	**Never**	**Former**	**Current**	**p**
**SBP**				0.27			0.99					0.60				0.62
No	121 (120-123)	127 (126-129)	133 (131-135)		124 (123-126)	129 (127-130)		126 (124-127)	129 (128-131)	124 (123-126)	127 (124-129)		126.64 (125-128)	126.21 (125-128)	128.86 (125-132)	
Yes	122 (119-125)	129 (123-135)	121 (105-136)		124 (121-128)	129 (124-134)		124 (120-128)	131 (127-135)	125 (117-133)	110 (71-148)		128.50 (122-135)	124.91 (122-128)	129.83 (123-137)	
**DBP**				0.82			0.55					0.94				0.40
No	70 (69-71)	68 (67-69)	67 (66-68)		70 (69-71)	67 (66-68)		67 (67-68)	71 (70-72)	68 (67-69)	68 (67-69)		68.74 (68-69)	68.26 (68-69)	68.28 (67-70)	
Yes	70 (69-72)	69 (66-72)	70 (62-77)		71 (69-72)	67 (64-69)		68 (66-70)	72 (70-74)	67 (64-71)	63 (45-82)		67.66 (65-71)	68.93 (67-71)	70.36 (67-74)	
**PP**				**0.04**			0.96					0.27				0.16
No	52 (50-53)	59 (58-60)	66 (64-67)		54 (53-55)	62 (61-63)		58 (57-59)	59 (57-60)	57 (55-58)	59 (57-60)		57.81 (57-59)	57.92 (57-59)	60.57 (58-63)	
Yes	51 (49-54)	60 (55-64)	50 (38-62)		53 (51-56)	62 (58-65)		56 (53-59)	60 (57-63)	59 (52-65)	44 (15-73)		60.70 (56-65)	55.74 (53-58)	59.60 (54-65)	

Model 1: age, gender, race/ethnicity, education, HH income.
Model 2: Model 1 plus alcohol and cigarette smoking, physical activity
Model 3 : Model 2 plus BMI, total cholesterol to HDL cholesterol ratio, FBG, antihypertensive, antilipidemic, and antidiabetic medications.
Abbreviations: CI, confidence interval; HH, household income; BMI, body mass index; HDL, high density lipoprotein; FBG, fasting blood glucose
Bold = p < 0.05

Table 3b (Supplementary) - Adjusted prevalence ratios and 95% confidence intervals for hypertension, stratified by selected characteristics

Characteristic	Age (n=2,824)				Sex (n=2,824)			Race/Ethnicity (n=2,824)					Tobacco Smoking Status (n=2,863)			
	59-69	70-80	> 80	p	Male	Female	p	Non-Hispanic White	Non-Hispanic Black	Hispanic	Chinese	p	Never	Former	Current	p
**History of regular cannabis smoking**	0.98 (0.80-1.20)	1.00 (0.80-1.24)	0.85 (0.69-1.06)	0.43	0.98 (0.80-1.19)	0.93 (0.75-1.16)	0.81	0.94 (0.77-1.15)	1.09 (0.87-1.37)	0.90 (0.73-1.12)	NM	0.08	1.12 (0.85-1.48)	1.01 (0.85-1.21)	1.05 (0.79-1.40)	0.79

Prevalence ratios and 95% CI for hypertension. Statistically significant PRs in bold.
Interaction p value calculated w/ one interaction term in model 3.
Model 3: age, gender, race/ethnicity, HH income, alcohol and cigarette smoking, physical activity, BMI, total cholesterol to HDL cholesterol ratio, FBG, antilipidemic, and antidiabetic medications.
Abbreviations: CI, confidence interval; HH, household income; BMI, body mass index; HDL, high density lipoprotein; FBG, fasting blood glucose
Bold = p < 0.10

Table 4 (Supplementary) – Adjusted beta coefficients for blood pressure for selected cannabis smoking characteristics

Characteristic	Unadjusted			Model 1			Model 2			Model 3		
	SBP	DBP	PP	SBP	DBP	PP	SBP	DBP	PP	SBP	DBP	PP
**Years of regular smoking (n=2,824)**	-0.1 (-0.3 to 0.0)	**0.1 (0.0 to 0.2)**	**-0.2 (-0.3 to -0.1)**	0.0 (-0.2 to 0.1)	0.0 (-0.1 to 0.1)	0.0 (-0.2 to 0.1)	0.0 (-0.1 to 0.2)	0.0 (-0.1 to 0.1)	0.0 (-0.1 to 0.1)	0.0 (-0.1 to 0.2)	0.0 (-0.1 to 0.1)	0.0 (-0.1 to 0.1)
**Joints/Pipes per day (n=2,824)**	-0.9 (-2.1 to 0.3)	**1.3 (0.7 to 1.9)**	**-2.2 (-3.2 to -1.2)**	0.1 (-1.1 to 1.3)	0.4 (-0.2 to 1.0)	-0.3 (-1.2 to 0.6)	0.3 (-1.2 to 1.8)	0.2 (-0.5 to 1.0)	0.1 (-1.1 to 1.2)	0.1 (-1.1 to 1.3)	0.3 (-0.3 to 0.8)	-0.1 (-1.0 to 0.8)
**Joint/Pipe years (n=2,815)**												
Low end of monthly frequency	0.0 (-0.1 to 0.1)	**0.0 (0.0 to 0.1)**	-0.1 (-0.1 to 0.0)	0.0 (-0.1 to 0.1)	0.0 (0.0 to 0.0)	0.0 (-0.1 to 0.1)	0.0 (-0.1 to 0.2)	0.0 (-0.1 to 0.0)	0.1 (0.0 to 0.2)	0.0 (-0.1 to 0.1)	0.0 (0.0 to 0.0)	0.0 (0.0 to 0.1)
Midpoint of monthly frequency	0.0 (-0.1 to 0.1)	**0.0 (0.0 to 0.1)**	**-0.1 (-0.1 to 0.0)**	0.0 (-0.1 to 0.1)	0.0 (0.0 to 0.0)	0.0 (-0.1 to 0.1)	0.0 (-0.1 to 0.1)	0.0 (-0.1 to 0.0)	0.0 (0.0 to 0.1)	0.0 (0.0 to 0.1)	0.0 (0.0 to 0.0)	0.0 (0.0 to 0.1)
High end of monthly frequency	0.0 (-0.1 to 0.0)	**0.0 (0.0 to 0.1)**	**-0.1 (-0.1 to 0.0)**	0.0 (-0.1 to 0.1)	0.0 (0.0 to 0.0)	0.0 (-0.1 to 0.0)	0.0 (-0.1 to 0.1)	0.0 (-0.1 to 0.0)	0.0 (0.0 to 0.1)	0.0 (0.0 to 0.1)	0.0 (0.0 to 0.0)	0.0 (0.0 to 0.1)

Low end, midpoint, and high end of each categorical response to "During the time that you smoked marijuana or hashish regularly, how often would you usually smoke it?".
Model 1: age, gender, race/ethnicity, education, HH income.
Model 2: Model 1 plus alcohol and cigarette use, physical activity
Model 3 : Model 2 plus BMI, total cholesterol to HDL cholesterol ratio, FBG, antihypertensive, antilipidemic, and antidiabetic medications.
Abbreviations: CI, confidence interval; HH, household; BMI, body mass index; HDL, high density lipoprotein; FBG, fasting blood glucose
Bold = p < 0.05

Table 5 (Supplementary) – Adjusted mean blood pressure and 95% confidence intervals among participants denying MI or stroke, by cannabis smoking status (subgroup, n=3,105)

Characteristic	Model 3		
	**SBP**	**DBP**	**PP**
**≥ 100 Joints/pipes lifetime (n=2,637)**			
No	126 (125-127)	69 (68-69)	58 (57-59)
Yes	126 (123-129)	69 (67-70)	57 (55-59)
**p**	0.89	0.99	0.74
**History of regular smoking (n=2,637)**			
No	126 (124-128)	70 (69-71)	57 (55-58)
Yes	128 (124-131)	70 (68-72)	57 (54-60)
**p**	0.49	0.77	0.64
**Duration of regular smoking, years (n=2,602)**			
0	126 (124-128)	70 (69-71)	57 (55-58)
1 to 5	128 (123-133)	69 (67-71)	59 (55-63)
> 5 to 10	125 (118-131)	67 (64-70)	57 (52-62)
> 10	127 (122-131)	69 (67-72)	57 (54-61)
**p**	0.84	0.77	0.89
**Frequency of regular smoking (n=2,628)**			
0 times a week	126 (124-128)	70 (69-71)	57 (55-58)
< Once a week	128 (121-135)	70 (67-73)	58 (52-63)
≥ Once a week	127 (123-131)	69 (67-71)	58 (55-61)
Daily	126 (121-132)	68 (66-71)	57 (53-62)
**p**	0.94	0.74	1.00
**Quantity of daily smoking (n=2,591)**			
0 joints/pipes	126 (124-128)	70 (69-71)	57 (55-58)
≤ 1 joints/pipes	129 (124-133)	71 (68-73)	57 (54-61)
1 to 3 joints/pipes	127 (122-132)	67 (65-70)	60 (55-64)
> 3 joints/pipes	126 (117-135)	71 (66-75)	55 (49-62)
**p**	0.73	0.11	0.73
**Recency of smoking (n=2,625)**			
No history of regular smoking	126 (124-128)	70 (69-71)	57 (55-58)
No past month smoking	126 (122-129)	68 (67-70)	57 (54-60)
Past month smoking	128 (123-132)	69 (67-72)	58 (54-62)
**p**	0.80	0.77	0.91

Model 3: Model 2 plus BMI, total cholesterol to HDL cholesterol ratio, FBG, antihypertensive, antilipidemic, and antidiabetic medications.
Abbreviations: CI, confidence interval; HH, household; BMI, body mass index; HDL, high density lipoprotein; FBG, fasting blood glucose
Bold = p < 0.05

Table 6 (Supplementary) - Adjusted prevalence ratios and 95% confidence intervals for hypertension among participants denying MI or stroke, by cannabis smoking status (subgroup, n=3,105)

**Characteristic**	**Model 3**
	**PR (95% CI)**
**≥ 100 Joints/pipes lifetime (n=2,637)**	
No	**1.00 Ref.**
Yes	1.00 (0.94-1.07)
**History of regular smoking (n=2,637)**	
No	**1.00 Ref.**
Yes	1.04 (0.97-1.11)
**Duration of regular smoking, years (n=2,602)**	
0	**1.00 Ref.**
1 to 5	1.06 (0.93-1.21)
> 5 to 10	1.05 (0.93-1.17)
> 10	0.99 (0.84-1.16)
**Frequency of regular smoking (n=2,628)**	
0 times a week	**1.00 Ref.**
< Once a week	1.00 (0.87-1.13)
≥ Once a week	1.07 (0.97-1.18)
Daily	1.01 (0.90-1.14)
**Quantity of daily smoking (n=2,591)**	
0 joints/pipes	**1.00 Ref.**
≤ 1 joints/pipes	1.05 (0.94-1.18)
> 1 joints/pipes	1.05 (0.94-1.17)
**Recency of smoking (n=2,625)**	
No history of regular smoking	**1.00 Ref.**
No past month smoking	1.01 (0.93-1.10)
Past month smoking	1.05 (0.93-1.19)

Model 3 : Model 2 plus BMI, total cholesterol to HDL cholesterol ratio, FBG, antilipidemic, and antidiabetic medications.
Abbreviations: CI, confidence interval; HH, household; BMI, body mass index; HDL, high density lipoprotein; FBG, fasting blood glucose
Bold = p < 0.05

Table 7 (Supplementary) – Adjusted mean blood pressure and 95% confidence intervals among participants not taking anti-hypertensive medications, by cannabis smoking status (subgroup, n=1,259)

Characteristic	Model 3		
	**SBP**	**DBP**	**PP**
**≥ 100 Joints/pipes lifetime (n=1,114)**			
No	123 (120-125)	68 (67-70)	54 (52-56)
Yes	121 (117-125)	68 (66-70)	53 (50-56)
**p**	0.52	0.52	0.59
**History of regular use (n=1,114)**			
No	122 (120-125)	68 (67-70)	54 (52-56)
Yes	122 (118-127)	68 (66-70)	54 (51-57)
**p**	0.89	0.75	0.94
**Duration of regular use, years (n=1,101)**			
0	122 (120-125)	68 (67-70)	54 (52-56)
1 to 5	124 (117-130)	68 (64-71)	55 (50-61)
> 5 to 10	120 (111-129)	68 (63-72)	52 (45-58)
> 10	123 (116-129)	68 (65-72)	54 (49-59)
**p**	0.94	0.96	0.84
**Frequency of regular use (n=1,111)**			
0 times a week	122 (120-125)	68 (67-70)	54 (52-56)
< Once a week	118 (107-130)	64 (58-70)	54 (46-63)
≥ Once a week	124 (118-129)	69 (67-72)	54 (50-58)
Daily	121 (113-129)	67 (63-71)	54 (48-59)
**p**	0.82	0.28	1.00
**Quantity of daily use (n=1,094)**			
0 joints/pipes	122 (120-125)	68 (67-70)	54 (52-56)
≤ 1 joints/pipes	123 (118-129)	69 (66-72)	53 (49-58)
> 1 to 3 joints/pipes	122 (115-129)	67 (63-70)	55 (50-61)
> 3 joints/pipes	126 (112-141)	70 (63-78)	55 (45-66)
**p**	0.92	0.57	0.90
**Recency of use (n=1,107)**			
No history of regular use	122 (120-125)	68 (67-70)	54 (52-56)
No past month use	121 (116-127)	68 (65-70)	53 (49-57)
Past month use	122 (115-129)	68 (65-71)	54 (49-59)
**p**	0.88	0.90	0.91

Model 3 : Model 2 plus BMI, total cholesterol to HDL cholesterol ratio, FBG, antilipidemic, and antidiabetic medications.
Abbreviations: CI, confidence interval; HH, household; BMI, body mass index; HDL, high density lipoprotein; FBG, fasting blood glucose
Bold = p < 0.05

Table 8 (Supplementary) - Adjusted prevalence ratios and 95% confidence intervals for hypertension among participants not taking anti-hypertensive medications, by cannabis smoking status (subgroup, n=1,259)

Characteristic	Model 3
	**PR (95% CI)**
**≥ 100 Joints/pipes lifetime (n=1,114)**	
No	**1.00 Ref.**
Yes	1.03 (0.79-1.35)
**History of regular use (n=1,114)**	
No	**1.00 Ref.**
Yes	1.15 (0.88-1.51)
**Duration of regular use, years (n=1,101)**	
0	**1.0 Ref.**
1 to 5	1.27 (0.83-1.96)
> 5 to 10	1.21 (0.82-1.79)
> 10	0.88 (0.47-1.63)
**Frequency of regular use (n=1,111)**	
0 times a week	**1.00 Ref.**
< Once a week	0.90 (0.37-2.17)
≥ Once a week	1.30 (0.94-1.79)
Daily	1.01 (0.61-1.65)
**Quantity of daily use (n=1,094)**	
0 joints/pipes	**1.00 Ref.**
≤ 1 joints/pipes	1.15 (0.79-1.69)
> 1 joints/pipes	1.27 (0.87-1.85)
**Recency of use (n=1,107)**	
No history of regular use	**1.00 Ref.**
No past month use	1.09 (0.77-1.54)
Past month use	1.15 (0.75-1.77)

Model 3 : Model 2 plus BMI, total cholesterol to HDL cholesterol ratio, FBG, antilipidemic, and antidiabetic medications.
Abbreviations: CI, confidence interval; HH, household; BMI, body mass index; HDL, high density lipoprotein; FBG, fasting blood glucose
Bold = $p < 0.05$

References

1. DE B, DA B, GL B, R D, AV DR, AR F, et al. Multi-Ethnic Study of Atherosclerosis: objectives and design. American journal of epidemiology. 2002;156(9).
